# Supplementary material for: Random forest as a generic framework for predictive modeling of spatial and spatio-temporal variables
Source: PeerJ. 2018 Aug 29;6:e5518. doi: 10.7717/peerj.5518 (PMC6119462; doi:10.7717/peerj.5518)
Supplement: Supplemental Information 1 [file peerj-06-5518-s001.pdf]

thengl / **GeoMLA**

Machine Learning algorithms for spatial and spatiotemporal data

Edit

Add topics

56 commits | **1** branch | **0** releases | **3** contributors | GPL-2.0

Branch: **master** ▾ | **New pull request** | **Create new file** | **Upload files** | **Find file** | **Clone or download** ▾

Tomislav Hengl Added locations of the data sets in the README — Latest commit `a304e88` 9 minutes ago

README_files	Added locations of the data sets in the README	9 minutes ago
RF_uncertainty	Keynote spatial accuracy	2 months ago
RF_vs_kriging	Install instructions	2 months ago
docs	Added license presentation eRum 2018	2 months ago
tex	Added locations of the data sets in the README	9 minutes ago
.gitignore	Presentation eRum 2018	2 months ago
GeoMLA.Rproj	Presentation eRum 2018	2 months ago
LICENSE	Presentation eRum 2018	2 months ago
README.R	Added slides Spatial Accuracy 2018	2 months ago
README.Rmd	Added locations of the data sets in the README	9 minutes ago
README.md	Added locations of the data sets in the README	9 minutes ago

README.md

RFsp — Random Forest for spatial data (R tutorial)

Hengl, T., Nussbaum, M., and Wright, M.N.

- Installing and loading packages
- Data sets in use
- Spatial prediction 2D continuous variable using buffer distances
- Spatial prediction 2D variable with covariates
- Spatial prediction of binomial variable
- Spatial prediction of categorical variable
- Spatial prediction of variables with extreme values
- Weighted RFsp
- Spatial prediction of multivariate problems
- Prediction of spatio-temporal variable
- References

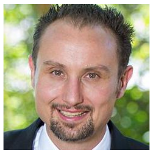 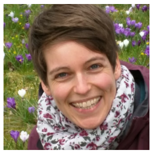 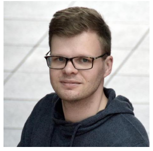

CC BY SA

Abstract: This tutorial explains how to use Random Forest to generate spatial and spatiotemporal predictions (i.e. to make maps from point observations using Random Forest). Spatial auto-correlation, especially if still existent in the cross-validation residuals, indicates that the predictions are maybe biased, and this is suboptimal. To account for this, we use Random Forest (as implemented in the ranger package) in combination with geographical distances to sampling locations to fit models and predict values. We describe eight typical situations of interest to spatial prediction applications: (1) prediction of 2D continuous variable without any covariates, (2) prediction of 2D variable with covariates, (3) prediction of binomial variable, (4) prediction of categorical variable, (5) prediction of variables with extreme values, (6) weighted regression, (7) predictions of multivariate problems, and (8) prediction of spatio-temporal variable. Our results indicate that RFsp can produce comparable results to model-based geostatistics. The advantage of RFsp over model-based geostatistics is that RFsp requires much less statistical assumptions and is easier to automate (and scale up through parallelization). On the other hand, computational intensity of RFsp can blow up as the number of training points and covariates increases. RFsp is still an experimental method and application with large data sets (>>1000 points) is not recommended. This is a supplementary material prepared for the need of a scientific article: Hengl, T., Nussbaum, M., Wright, M. and Heuvelink, G.B.M., 2018. *"Random Forest as a Generic Framework for Predictive Modeling of Spatial and Spatio-temporal Variables"*, PeerJ (in review). To download all data sets and more detail code examples please refer to https://github.com/thengl/GeoMLA/tree/master/RF_vs_kriging

Installing and loading packages

To run this tutorial it is recommended to install ranger (Wright & Ziegler, 2017) directly from github:

```
devtools::install_github("imbs-hl/ranger")
```

Quantile regression random forest and derivation of standard errors using Jackknifing is available from ranger version >0.9.4. Other packages that we use here include:

```
library(GSIF)
```

```
## GSIF version 0.5-4 (2017-04-25)

## URL: http://gsif.r-forge.r-project.org/
```

```
library(rgdal)
```

```
## Loading required package: sp

## rgdal: version: 1.3-3, (SVN revision (unknown))
##  Geospatial Data Abstraction Library extensions to R successfully loaded
##  Loaded GDAL runtime: GDAL 2.3.1, released 2018/06/22
##  Path to GDAL shared files: /usr/local/share/gdal
##  GDAL binary built with GEOS: TRUE
##  Loaded PROJ.4 runtime: Rel. 4.9.2, 08 September 2015, [PJ_VERSION: 492]
##  Path to PROJ.4 shared files: (autodetected)
##  Linking to sp version: 1.2-5
```

```
library(raster)
library(geoR)
```

```
## --------------------------------------------------------------
##  Analysis of Geostatistical Data
##  For an Introduction to geoR go to http://www.leg.ufpr.br/geoR
```

```
##  geoR version 1.7-5.2 (built on 2016-05-02) is now loaded
## --------------------------------------------------------------
```

```
library(ranger)
```

```
library(gstat)
library(intamap)
```

```
##
## Attaching package: 'intamap'

## The following object is masked from 'package:raster':
##
##     interpolate
```

```
library(plyr)
library(plotKML)
```

```
## plotKML version 0.5-9 (2017-05-15)

## URL: http://plotkml.r-forge.r-project.org/
```

```
library(scales)
library(RCurl)
```

```
## Loading required package: bitops
```

```
library(parallel)
library(lattice)
library(gridExtra)
```

We also load a number of local function prepared for the purpose of this tutorial:

```
source('./RF_vs_kriging/R/RFsp_functions.R')
```

Data sets in use

This tutorial uses several data sets that are either available from the R packages listed or can be loaded from the `/RF_vs_kriging/data` directory. This is the complete list of data sets used in the tutorial and the scientific paper:

- Muse data set (sp package): topsoil heavy metal concentrations, along with a number of soil and landscape variables at 155 observation locations;
- Swiss rainfall dataset SIC 1997 (G Dubois, Malczewski, & De Cort, 2003) (see folder `./RF_vs_kriging/data/rainfall`): 467 measurements of daily rainfall in Switzerland on the 8th of May 1986;
- Ebergötzen data set (plotKML package): 3670 ground observations of soil types and texture by hand,
- National Cooperative Soil Survey (NCSS) data set for a sub-area around Carson (see folder `./RF_vs_kriging/data/NRCS`): contains 3418 measurements of clay content in soil;
- The National Geochemical Survey data set (see folder `./RF_vs_kriging/data/geochem`): 2858 points with measurements of Pb, Cu, K and Mg covering the US states Illinois and Indiana;

- Boulder Colorado daily precipitation (see folder `./RF_vs_kriging/data/st_prec`): 176,467 measurements of daily precipitation for the period 20142017,

Spatial prediction 2D continuous variable using buffer distances

If no other information is available, we can use buffer distances to all points as covariates to predict values of some continuous or categorical variable in the RFsp framework. These can be derived with the help of the raster package (Hijmans & Etten, 2017). Consider for example the meuse data set from the gstat package:

```
demo(meuse, echo=FALSE)
```

We can derive buffer distance by using:

```
grid.dist0 <- GSIF::buffer.dist(meuse["zinc"], meuse.grid[1], as.factor(1:nrow(meuse)))
```

which takes few seconds as it generates 155 gridded maps. The value of the target variable `zinc` can be now modeled as a function of buffer distances:

```
dn0 <- paste(names(grid.dist0), collapse="+")
fm0 <- as.formula(paste("zinc ~ ", dn0))
fm0
```

```
## zinc ~ layer.1 + layer.2 + layer.3 + layer.4 + layer.5 + layer.6 + 
##     layer.7 + layer.8 + layer.9 + layer.10 + layer.11 + layer.12 + 
##     layer.13 + layer.14 + layer.15 + layer.16 + layer.17 + layer.18 + 
##     layer.19 + layer.20 + layer.21 + layer.22 + layer.23 + layer.24 + 
##     layer.25 + layer.26 + layer.27 + layer.28 + layer.29 + layer.30 + 
##     layer.31 + layer.32 + layer.33 + layer.34 + layer.35 + layer.36 + 
##     layer.37 + layer.38 + layer.39 + layer.40 + layer.41 + layer.42 + 
##     layer.43 + layer.44 + layer.45 + layer.46 + layer.47 + layer.48 + 
##     layer.49 + layer.50 + layer.51 + layer.52 + layer.53 + layer.54 + 
##     layer.55 + layer.56 + layer.57 + layer.58 + layer.59 + layer.60 + 
##     layer.61 + layer.62 + layer.63 + layer.64 + layer.65 + layer.66 + 
##     layer.67 + layer.68 + layer.69 + layer.70 + layer.71 + layer.72 + 
##     layer.73 + layer.74 + layer.75 + layer.76 + layer.77 + layer.78 + 
##     layer.79 + layer.80 + layer.81 + layer.82 + layer.83 + layer.84 + 
##     layer.85 + layer.86 + layer.87 + layer.88 + layer.89 + layer.90 + 
##     layer.91 + layer.92 + layer.93 + layer.94 + layer.95 + layer.96 + 
##     layer.97 + layer.98 + layer.99 + layer.100 + layer.101 + 
##     layer.102 + layer.103 + layer.104 + layer.105 + layer.106 + 
##     layer.107 + layer.108 + layer.109 + layer.110 + layer.111 + 
##     layer.112 + layer.113 + layer.114 + layer.115 + layer.116 + 
##     layer.117 + layer.118 + layer.119 + layer.120 + layer.121 + 
##     layer.122 + layer.123 + layer.124 + layer.125 + layer.126 + 
##     layer.127 + layer.128 + layer.129 + layer.130 + layer.131 + 
##     layer.132 + layer.133 + layer.134 + layer.135 + layer.136 + 
##     layer.137 + layer.138 + layer.139 + layer.140 + layer.141 + 
##     layer.142 + layer.143 + layer.144 + layer.145 + layer.146 + 
##     layer.147 + layer.148 + layer.149 + layer.150 + layer.151 + 
##     layer.152 + layer.153 + layer.154 + layer.155
```

Further analysis is similar to any regression analysis using the ranger package. First we overlay points and grids to create a regression matrix:

```
ov.zinc <- over(meuse["zinc"], grid.dist0)
rm.zinc <- cbind(meuse@data["zinc"], ov.zinc)
```

to estimate also the prediction error variance i.e. prediction intervals we set `quantreg=TRUE` which initiates the Quantile Regression RF approach (Meinshausen, 2006):

```
m.zinc <- ranger(fm0, rm.zinc, quantreg=TRUE, num.trees=150, seed=1)
m.zinc
```

```
## Ranger result
##
## Call:
##  ranger(fm0, rm.zinc, quantreg = TRUE, num.trees = 150, seed = 1)
##
## Type:                             Regression
## Number of trees:                  150
## Sample size:                      155
## Number of independent variables:  155
## Mtry:                             12
## Target node size:                 5
## Variable importance mode:         none
## OOB prediction error (MSE):       67501.48
## R squared (OOB):                  0.4990359
```

This shows that, only buffer distance explain almost 50% of variation in the target variable. To generate prediction for the `zinc` variable and using the RFsp model, we use:

```
zinc.rfd <- predict(m.zinc, grid.dist0@data, type="quantiles", quantiles=quantiles)$predictions
str(zinc.rfd)
```

```
##  num [1:3103, 1:3] 257 257 257 257 257 ...
##  - attr(*, "dimnames")=List of 2
##   ..$ : NULL
##   ..$ : chr [1:3] "quantile= 0.159" "quantile= 0.5" "quantile= 0.841"
```

this will estimate 67% probability lower and upper limits and median value. Note that "median" can often be different from the "mean", so if you prefer to derive mean, then the `quantreg=FALSE` needs to be used as the Quantile Regression Forests approach can only derive median.

To be able to plot or export predicted values as maps, we add them to the spatial pixels object:

```
meuse.grid$zinc_rfd = zinc.rfd[,2]
meuse.grid$zinc_rfd_range = (zinc.rfd[,3]-zinc.rfd[,1])/2
```

We can compare the RFsp approach with the model-based geostatistics (see e.g. geoR package), where we first decide about the transformation, then fit variogram of the target variable (Brown, 2015; Diggle & Ribeiro Jr, 2007):

```
zinc.geo <- as.geodata(meuse["zinc"])
ini.v <- c(var(log1p(zinc.geo$data)),500)
zinc.vgm <- likfit(zinc.geo, lambda=0, ini=ini.v, cov.model="exponential")
```

```
## kappa not used for the exponential correlation function
## ---------------------------------------------------------------
## likfit: likelihood maximisation using the function optim.
## likfit: Use control() to pass additional
##          arguments for the maximisation function.
##         For further details see documentation for optim.
## likfit: It is highly advisable to run this function several
##         times with different initial values for the parameters.
## likfit: WARNING: This step can be time demanding!
## ---------------------------------------------------------------
## likfit: end of numerical maximisation.
```

```
zinc.vgm
```

```
## likfit: estimated model parameters:
##       beta      tausq    sigmasq        phi 
## "  6.1553" "  0.0164" "  0.5928" "500.0001" 
## Practical Range with cor=0.05 for asymptotic range: 1497.866
## 
## likfit: maximised log-likelihood = -1014
```

where `likfit` function fits log-likelihood based variogram. Note that we here manually need to specify log-transformation via the `lambda` parameter. To generate predictions and kriging variance using geoR we run:

```
locs = meuse.grid@coords
zinc.ok <- krige.conv(zinc.geo, locations=locs, krige=krige.control(obj.model=zinc.vgm))
```

```
## krige.conv: model with constant mean
## krige.conv: performing the Box-Cox data transformation
## krige.conv: back-transforming the predicted mean and variance
## krige.conv: Kriging performed using global neighbourhood
```

```
meuse.grid$zinc_ok = zinc.ok$predict
meuse.grid$zinc_ok_range = sqrt(zinc.ok$krige.var)
```

in this case geoR automatically back-transforms values to the original scale, which is a recommended feature. Comparison of predictions and prediction error maps produced using geoR (ordinary kriging) and RFsp (with buffer distances and by just using coordinates) is given below.

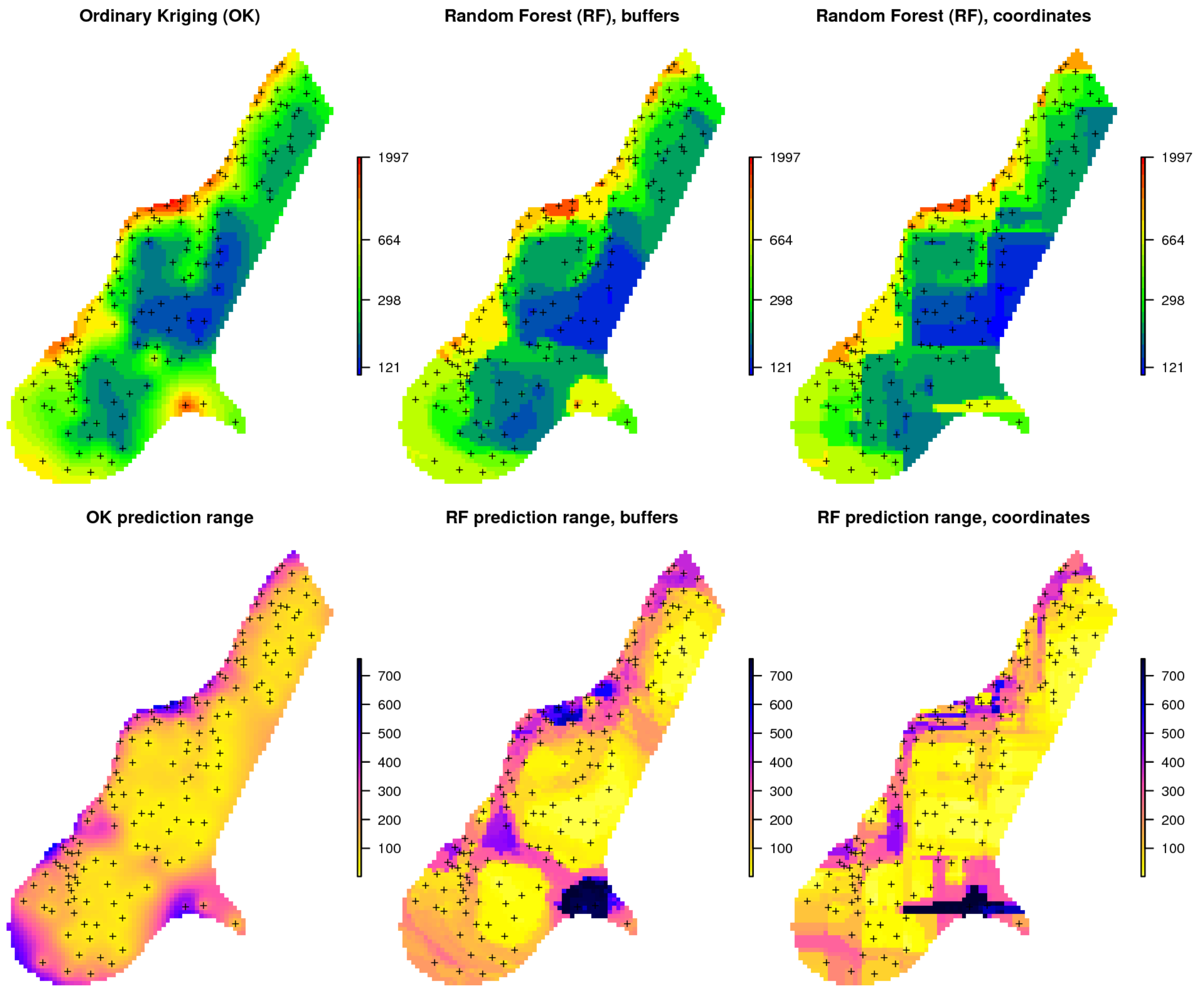

Figure: Comparison of predictions based on ordinary kriging as implemented in the geoR package (left) and random forest (right) for Zinc concentrations, Meuse data set: (first row) predicted concentrations in log-scale and (second row) standard deviation of the prediction errors for OK and RF methods.

From the plot above, it can be concluded that RFsp gives very similar results as ordinary kriging via geoR. The differences between geoR and RFsp, however, are:

- RF requires no transformation i.e. works equally good with skewed and normally distributed variables; in general RF, has much less statistical assumptions than model-based geostatistics,
- RF prediction error variance in average shows somewhat stronger contrast than OK variance map i.e. it emphasizes isolated less probable local points much more than geoR,
- RFsp is significantly more computational as distances need to be derived from any sampling point to all new predictions locations,
- geoR uses global model parameters and as such also prediction patterns are relatively uniform, RFsp on the other hand (being a tree-based) will produce patterns that as much as possible match data,

Spatial prediction 2D variable with covariates

Next we can also consider adding extra covariates that describe soil forming processes or characteristics of the land of interest to the list of buffer distances, for example the surface water occurrence (Pekel, Cottam, Gorelick, & Belward, 2016) and elevation (AHN):

```
meuse.grid$SW_occurrence = readGDAL("./RF_vs_kriging/data/meuse/Meuse_GlobalSurfaceWater_occurrence.tif")$ba
```

```
## ./RF_vs_kriging/data/meuse/Meuse_GlobalSurfaceWater_occurrence.tif has GDAL driver GTiff
## and has 104 rows and 78 columns
```

```
meuse.grid$AHN = readGDAL("./RF_vs_kriging/data/meuse/ahn.asc")$band1[meuse.grid@grid.index]
```

```
## ./RF_vs_kriging/data/meuse/ahn.asc has GDAL driver AAIGrid
## and has 104 rows and 78 columns
```

to convert all covariates to numeric values and impute all missing pixels we use Principal Component transformation:

```
grids.spc = GSIF::spc(meuse.grid, as.formula("~ SW_occurrence + AHN + ffreq + dist"))
```

```
## Converting ffreq to indicators...

## Converting covariates to principal components...
```

so that we can fit a ranger model using both geographical covariates (buffer distances) and covariates imported previously:

```
fm1 <- as.formula(paste("zinc ~ ", dn0, " + ", paste(names(grids.spc@predicted), collapse = "+")))
fm1
```

```
## zinc ~ layer.1 + layer.2 + layer.3 + layer.4 + layer.5 + layer.6 +
##     layer.7 + layer.8 + layer.9 + layer.10 + layer.11 + layer.12 +
##     layer.13 + layer.14 + layer.15 + layer.16 + layer.17 + layer.18 +
##     layer.19 + layer.20 + layer.21 + layer.22 + layer.23 + layer.24 +
##     layer.25 + layer.26 + layer.27 + layer.28 + layer.29 + layer.30 +
##     layer.31 + layer.32 + layer.33 + layer.34 + layer.35 + layer.36 +
##     layer.37 + layer.38 + layer.39 + layer.40 + layer.41 + layer.42 +
##     layer.43 + layer.44 + layer.45 + layer.46 + layer.47 + layer.48 +
##     layer.49 + layer.50 + layer.51 + layer.52 + layer.53 + layer.54 +
##     layer.55 + layer.56 + layer.57 + layer.58 + layer.59 + layer.60 +
##     layer.61 + layer.62 + layer.63 + layer.64 + layer.65 + layer.66 +
##     layer.67 + layer.68 + layer.69 + layer.70 + layer.71 + layer.72 +
##     layer.73 + layer.74 + layer.75 + layer.76 + layer.77 + layer.78 +
##     layer.79 + layer.80 + layer.81 + layer.82 + layer.83 + layer.84 +
##     layer.85 + layer.86 + layer.87 + layer.88 + layer.89 + layer.90 +
##     layer.91 + layer.92 + layer.93 + layer.94 + layer.95 + layer.96 +
##     layer.97 + layer.98 + layer.99 + layer.100 + layer.101 +
##     layer.102 + layer.103 + layer.104 + layer.105 + layer.106 +
##     layer.107 + layer.108 + layer.109 + layer.110 + layer.111 +
##     layer.112 + layer.113 + layer.114 + layer.115 + layer.116 +
##     layer.117 + layer.118 + layer.119 + layer.120 + layer.121 +
##     layer.122 + layer.123 + layer.124 + layer.125 + layer.126 +
##     layer.127 + layer.128 + layer.129 + layer.130 + layer.131 +
##     layer.132 + layer.133 + layer.134 + layer.135 + layer.136 +
##     layer.137 + layer.138 + layer.139 + layer.140 + layer.141 +
##     layer.142 + layer.143 + layer.144 + layer.145 + layer.146 +
##     layer.147 + layer.148 + layer.149 + layer.150 + layer.151 +
##     layer.152 + layer.153 + layer.154 + layer.155 + PC1 + PC2 +
##     PC3 + PC4 + PC5 + PC6
```

```
ov.zinc1 <- over(meuse["zinc"], grids.spc@predicted)
rm.zinc1 <- do.call(cbind, list(meuse@data["zinc"], ov.zinc, ov.zinc1))
```

this finally gives:

```r
m1.zinc <- ranger(fm1, rm.zinc1, importance="impurity", quantreg=TRUE, num.trees=150, seed=1)
m1.zinc
```

```
## Ranger result
## 
## Call:
##  ranger(fm1, rm.zinc1, importance = "impurity", quantreg = TRUE,      num.trees = 150, seed = 1) 
## 
## Type:                             Regression 
## Number of trees:                  150 
## Sample size:                      155 
## Number of independent variables:  161 
## Mtry:                             12 
## Target node size:                 5 
## Variable importance mode:         impurity 
## OOB prediction error (MSE):       54749.82 
## R squared (OOB):                  0.5936727
```

there is a slight improvement from using only buffer distances as covariates. We can further evaluate this model to see which specific points and covariates are most important for spatial predictions:

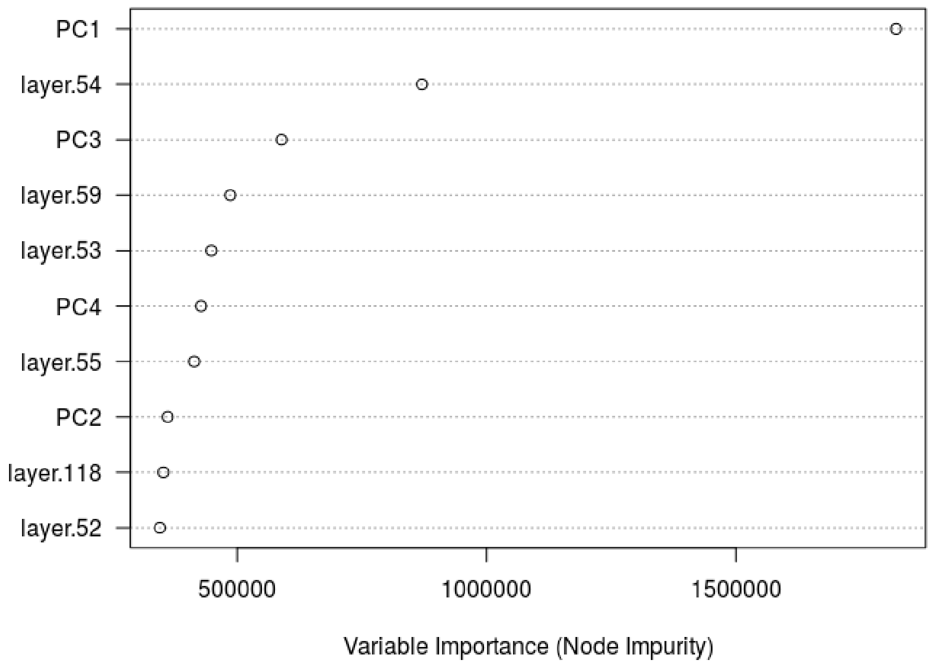

which shows, for example, that point 54 and 53 are two most influential points, and these are almost equally important as covariates (PC2--PC4).

This type of modeling can be best compared to using Universal Kriging or Regression-Kriging in the geoR package:

```r
zinc.geo$covariate = ov.zinc1
sic.t = ~ PC1 + PC2 + PC3 + PC4 + PC5
zinc1.vgm <- likfit(zinc.geo, trend = sic.t, lambda=0, ini=ini.v, cov.model="exponential")
```

```
## kappa not used for the exponential correlation function
## ---------------------------------------------------------------
## likfit: likelihood maximisation using the function optim.
## likfit: Use control() to pass additional
##          arguments for the maximisation function.
##         For further details see documentation for optim.
## likfit: It is highly advisable to run this function several
##         times with different initial values for the parameters.
```

```
## likfit: WARNING: This step can be time demanding!
## ---------------------------------------------------------------
## likfit: end of numerical maximisation.
```

```
zinc1.vgm
```

```
## likfit: estimated model parameters:
##      beta0      beta1      beta2      beta3      beta4      beta5 
## "  5.6929" " -0.4351" "  0.0002" " -0.0791" " -0.0485" " -0.3725" 
##      tausq    sigmasq        phi 
## "  0.0566" "  0.1992" "499.9990" 
## Practical Range with cor=0.05 for asymptotic range: 1497.863
## 
## likfit: maximised log-likelihood = -979.5
```

this time geostatistical modeling results in estimate of beta (regression coefficients) and variogram parameters (all estimated at once). Predictions using this Universal Kriging model can be generated by:

```
KC = krige.control(trend.d = sic.t, trend.l = ~ grids.spc@predicted$PC1 + grids.spc@predicted$PC2 + grids.sp
zinc.uk <- krige.conv(zinc.geo, locations=locs, krige=KC)
```

```
## krige.conv: model with mean defined by covariates provided by the user
## krige.conv: performing the Box-Cox data transformation
## krige.conv: back-transforming the predicted mean and variance
## krige.conv: Kriging performed using global neighbourhood
```

```
meuse.grid$zinc_UK = zinc.uk$predict
```

again, overall predictions looks fairly similar. The difference between using geoR and RFsp is that, in the case of RFsp there are less choices and less assumptions to be made. Also, RFsp allows that relationship with covariates and geographical distances is fitted all at once. This makes RFsp in general less cumbersome than model-based geostatistics, but then more of a "black-box" system to a geostatistician.

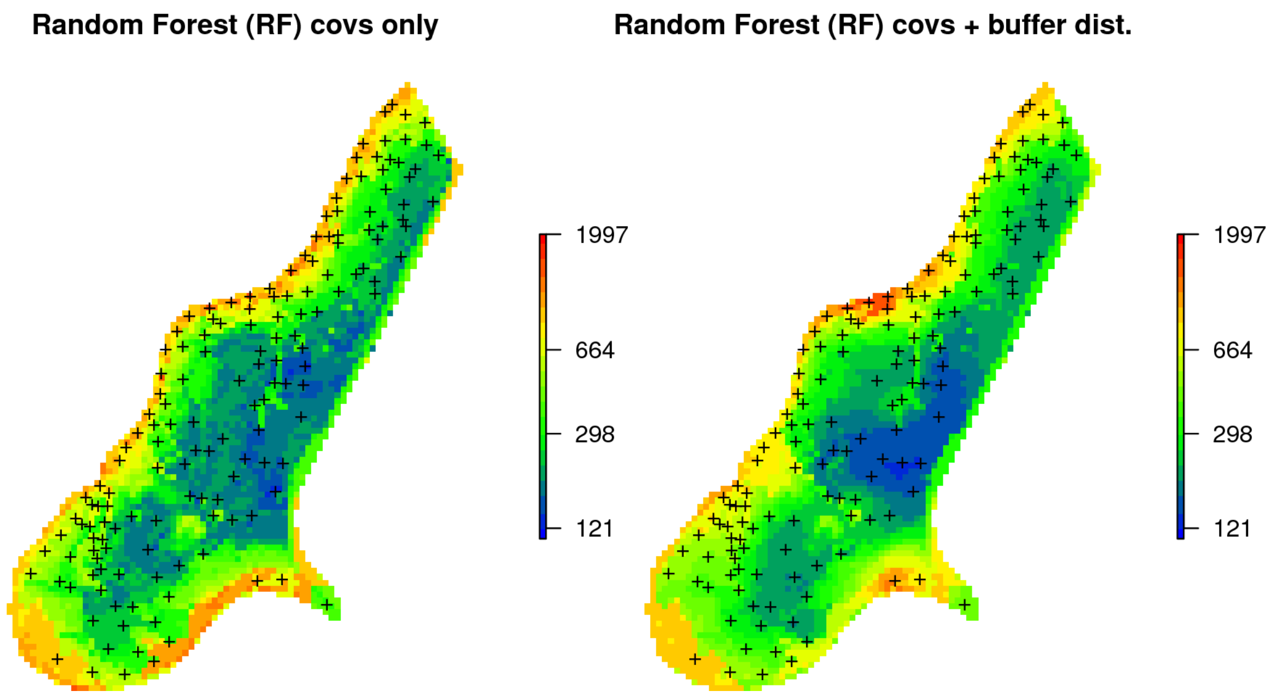

Figure: Comparison of predictions (median values) produced using random forest and covariates only (left), and random forest with combined covariates and buffer distances (right).

Spatial prediction of binomial variable

RFsp can also be used to predict i.e. map distribution of binomial variables i.e. having only two states (TRUE or FALSE). In the model-based geostatistics equivalent methods are indicator kriging and similar. Consider for example the soil type 1 from the meuse data set:

```
meuse@data = cbind(meuse@data, data.frame(model.matrix(~soil-1, meuse@data)))
summary(as.factor(meuse$soil1))
```

```
##  0  1
## 58 97
```

in this case class `soil1` is the dominant soil type in the area. To produce a map of `soil1` using RFsp we have now two options:

- *Option 1*: treat binomial variable as numeric variable with 0 / 1 values (thus a regression problem),
- *Option 2*: treat binomial variable as factor variable with a single class (thus a classification problem),

In the case of Option 1, we model `soil1` as:

```
fm.s1 = as.formula(paste("soil1 ~ ", paste(names(grid.dist0), collapse="+"), " + SW_occurrence + dist"))
rm.s1 <- do.call(cbind, list(meuse@data["soil1"], over(meuse["soil1"], meuse.grid), over(meuse["soil1"], gri
m1.s1 <- ranger(fm.s1, rm.s1, mtry=22, num.trees=150, seed=1, quantreg=TRUE)
m1.s1
```

```
## Ranger result
##
## Call:
##  ranger(fm.s1, rm.s1, mtry = 22, num.trees = 150, seed = 1, quantreg = TRUE)
##
## Type:                             Regression
```

```
## Number of trees:                 150
## Sample size:                     155
## Number of independent variables:  157
## Mtry:                            22
## Target node size:                5
## Variable importance mode:        none
## OOB prediction error (MSE):      0.05787064
## R squared (OOB):                 0.7544664
```

which shows that the model explains 75% of variability in the `soil1` values. We set `quantreg=TRUE` so that we can also derive lower and upper prediction intervals following the quantile regression random forest (Meinshausen, 2006).

In the case of Option 2, we treat binomial variable as a factor variable:

```
fm.s1c <- as.formula(paste("soil1c ~ ", paste(names(grid.dist0), collapse="+"), " + SW_occurrence + dist"))
rm.s1$soil1c = as.factor(rm.s1$soil1)
m2.s1 <- ranger(fm.s1c, rm.s1, mtry=22, num.trees=150, seed=1, probability=TRUE, keep.inbag=TRUE)
m2.s1
```

```
## Ranger result
##
## Call:
##  ranger(fm.s1c, rm.s1, mtry = 22, num.trees = 150, seed = 1, probability = TRUE,      keep.inbag = 
TRUE)
##
## Type:                            Probability estimation
## Number of trees:                 150
## Sample size:                     155
## Number of independent variables:  157
## Mtry:                            22
## Target node size:                10
## Variable importance mode:        none
## OOB prediction error:            0.05860483
```

which shows that the Out of Bag prediction error (classification error) is only 0.06 (in the probability scale). Note that, it is not easy to compare the results of the regression and classification OOB errors as these are conceptually different. Also note that we turn on `keep.inbag = TRUE` so that ranger can estimate the classification errors using the Jackknife-after-Bootstrap method (Wager, Hastie, & Efron, 2014). `quantreg=TRUE` obviously would not work here since it is a classification and not a regression problem.

To produce predictions using the two options we use:

```
pred.regr <- predict(m1.s1, cbind(meuse.grid@data, grid.dist0@data), type="response")
pred.clas <- predict(m2.s1, cbind(meuse.grid@data, grid.dist0@data), type="se")
```

in principle, the two options to predicting distribution of binomial variable are mathematically equivalent and should lead to same predictions (also shown in the map below). In practice there can be some smaller differences in numbers due to rounding effect or random start effects.

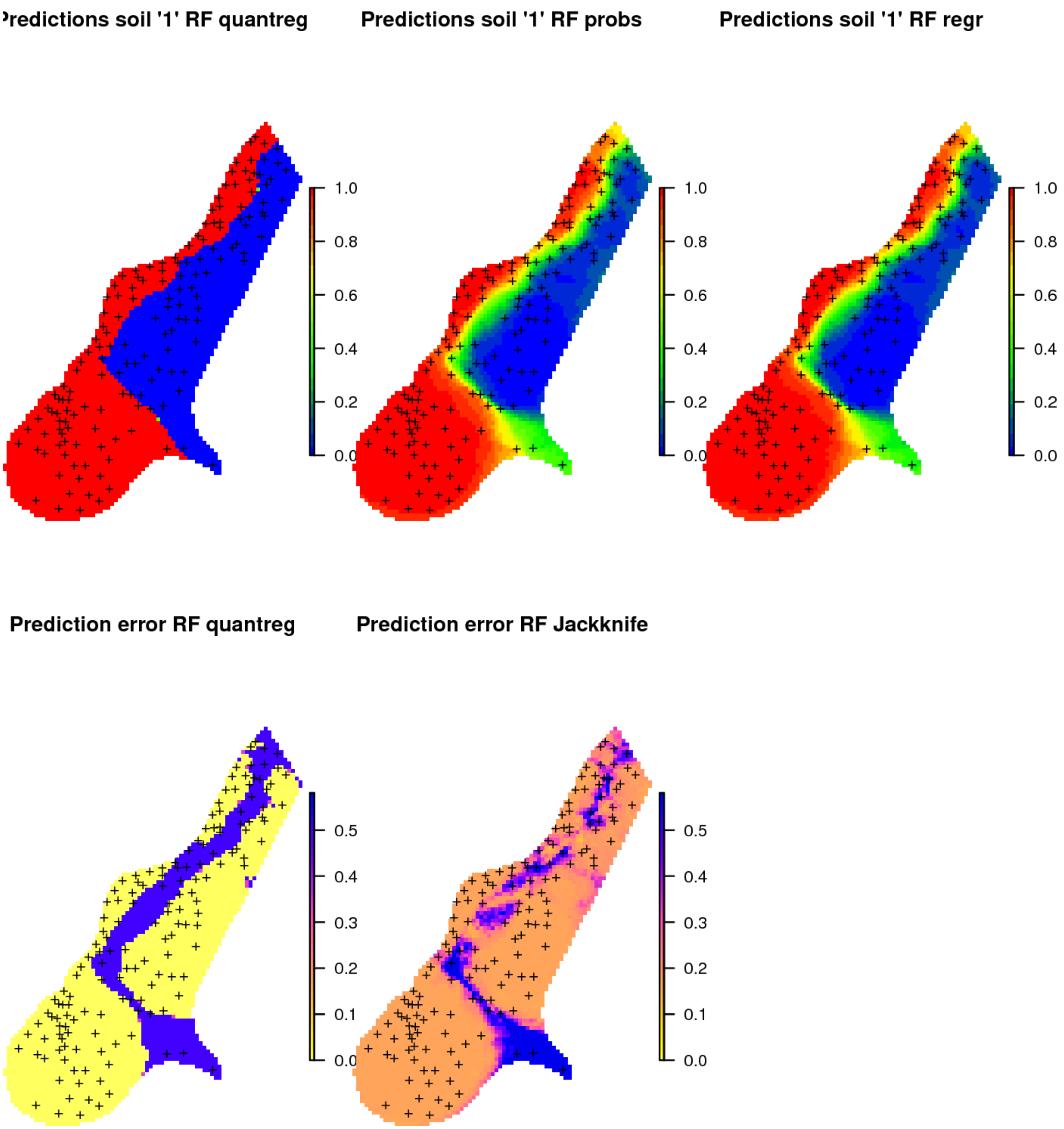

Figure: Comparison of predictions for soil class "1" produced using (left) regression and prediction of the median value, (middle) regression and prediction of response value, and (right) classification with probabilities.

In summary, predicting binomial variables using RFsp can be implemented both as a classification and regression problems and both are possible via the ranger package and should lead to same results.

Spatial prediction of categorical variable

Spatial prediction of categorical variable using ranger belongs to classification problems. The target variable contains multiple states (3 in this case), but the model follows still the same formulation:

```
fm.s = as.formula(paste("soil ~ ", paste(names(grid.dist0), collapse="+"), " + SW_occurrence + dist"))
fm.s
```

```
## soil ~ layer.1 + layer.2 + layer.3 + layer.4 + layer.5 + layer.6 +
##     layer.7 + layer.8 + layer.9 + layer.10 + layer.11 + layer.12 +
##     layer.13 + layer.14 + layer.15 + layer.16 + layer.17 + layer.18 +
##     layer.19 + layer.20 + layer.21 + layer.22 + layer.23 + layer.24 +
##     layer.25 + layer.26 + layer.27 + layer.28 + layer.29 + layer.30 +
##     layer.31 + layer.32 + layer.33 + layer.34 + layer.35 + layer.36 +
##     layer.37 + layer.38 + layer.39 + layer.40 + layer.41 + layer.42 +
##     layer.43 + layer.44 + layer.45 + layer.46 + layer.47 + layer.48 +
##     layer.49 + layer.50 + layer.51 + layer.52 + layer.53 + layer.54 +
##     layer.55 + layer.56 + layer.57 + layer.58 + layer.59 + layer.60 +
##     layer.61 + layer.62 + layer.63 + layer.64 + layer.65 + layer.66 +
##     layer.67 + layer.68 + layer.69 + layer.70 + layer.71 + layer.72 +
##     layer.73 + layer.74 + layer.75 + layer.76 + layer.77 + layer.78 +
##     layer.79 + layer.80 + layer.81 + layer.82 + layer.83 + layer.84 +
##     layer.85 + layer.86 + layer.87 + layer.88 + layer.89 + layer.90 +
##     layer.91 + layer.92 + layer.93 + layer.94 + layer.95 + layer.96 +
##     layer.97 + layer.98 + layer.99 + layer.100 + layer.101 +
##     layer.102 + layer.103 + layer.104 + layer.105 + layer.106 +
##     layer.107 + layer.108 + layer.109 + layer.110 + layer.111 +
##     layer.112 + layer.113 + layer.114 + layer.115 + layer.116 +
##     layer.117 + layer.118 + layer.119 + layer.120 + layer.121 +
##     layer.122 + layer.123 + layer.124 + layer.125 + layer.126 +
##     layer.127 + layer.128 + layer.129 + layer.130 + layer.131 +
##     layer.132 + layer.133 + layer.134 + layer.135 + layer.136 +
##     layer.137 + layer.138 + layer.139 + layer.140 + layer.141 +
##     layer.142 + layer.143 + layer.144 + layer.145 + layer.146 +
##     layer.147 + layer.148 + layer.149 + layer.150 + layer.151 +
##     layer.152 + layer.153 + layer.154 + layer.155 + SW_occurrence +
##     dist
```

to produce probability maps per soil class, we need to turn the `probability=TRUE` option:

```
rm.s <- do.call(cbind, list(meuse@data["soil"], over(meuse["soil"], meuse.grid), over(meuse["soil"], grid.di
m.s <- ranger(fm.s, rm.s, mtry=22, num.trees=150, seed=1, probability=TRUE, keep.inbag=TRUE)
m.s
```

```
## Ranger result
##
## Call:
##  ranger(fm.s, rm.s, mtry = 22, num.trees = 150, seed = 1, probability = TRUE,      keep.inbag = TRUE)
##
## Type:                             Probability estimation
## Number of trees:                  150
## Sample size:                      155
## Number of independent variables:  157
## Mtry:                             22
## Target node size:                 10
## Variable importance mode:         none
## OOB prediction error:             0.09217582
```

this shows that the model is succesful with the OOB prediction error of about 0.09. This number is rather abstract so we can also check what is the actual classification accuracy using hard classes:

```
m.s0 <- ranger(fm.s, rm.s, mtry=22, num.trees=150, seed=1)
m.s0
```

```
## Ranger result
##
## Call:
##  ranger(fm.s, rm.s, mtry = 22, num.trees = 150, seed = 1)
##
```

```
## Type:                             Classification
## Number of trees:                  150
## Sample size:                      155
## Number of independent variables:  157
## Mtry:                             22
## Target node size:                 1
## Variable importance mode:         none
## OOB prediction error:             10.32 %
```

which shows that the classification or mapping accuracy for hard classes is about 90%. We can produce predictions of probabilities per class by:

```
pred.soil_rfc = predict(m.s, cbind(meuse.grid@data, grid.dist0@data), type="se")
pred.grids = meuse.grid["soil"]
pred.grids@data = do.call(cbind, list(pred.grids@data, data.frame(pred.soil_rfc$predictions), data.frame(pre
names(pred.grids) = c("soil", paste0("pred_soil", 1:3), paste0("se_soil", 1:3))
str(pred.grids@data)
```

```
## 'data.frame':    3103 obs. of  7 variables:
##  $ soil      : Factor w/ 3 levels "1","2","3": 1 1 1 1 1 1 1 1 1 1 ...
##  $ pred_soil1: num  0.716 0.713 0.713 0.693 0.713 ...
##  $ pred_soil2: num  0.246 0.256 0.256 0.27 0.256 ...
##  $ pred_soil3: num  0.0374 0.0307 0.0307 0.0374 0.0307 ...
##  $ se_soil1  : num  0.181 0.1695 0.1695 0.0895 0.1695 ...
##  $ se_soil2  : num  0.1435 0.081 0.081 0.0798 0.081 ...
##  $ se_soil3  : num  0.0393 0.0392 0.0392 0.0393 0.0392 ...
```

where `pred_soil1` is the probability of occurrence of class 1 and `se_soil1` is the standard error of prediction for the `pred_soil1` based on the Jackknife-after-Bootstrap method (Wager et al., 2014). The first column in `pred.grids` contains existing map of `soil` with hard classes only.

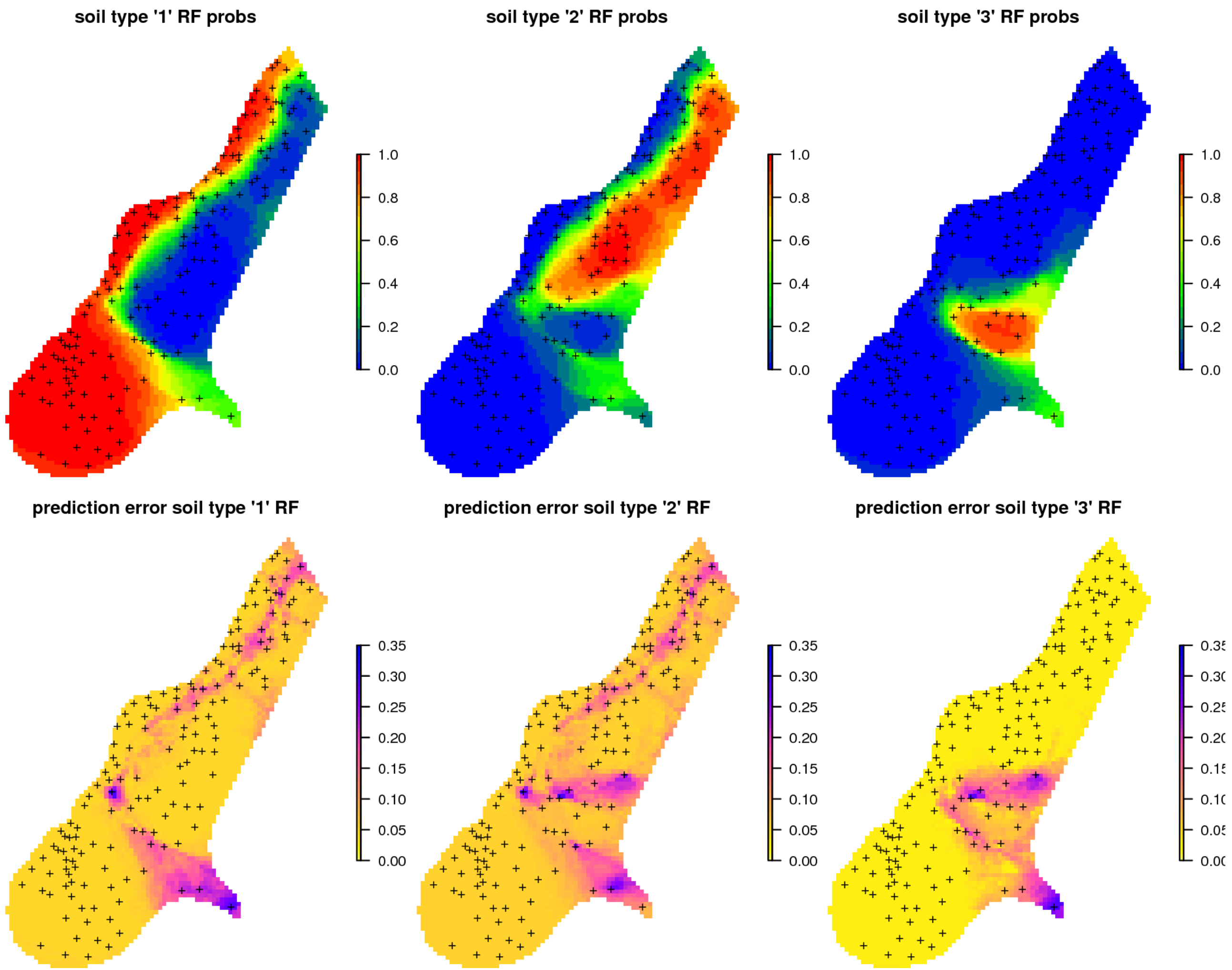

Figure: Predictions of soil types for the meuse data set based on the RFsp: (above) probability for three soil classes, and (below) derived standard errors per class.

In summary, spatial prediction of binomial and factor-type variables is straight forward with ranger: buffer distance and spatial-autocorrelation can be incorporated at once. Compare with geostatistical packages where GLMs with logit link function and/or indicator kriging would need to be used, and which requires that variograms are fitted per class.

Spatial prediction of variables with extreme values

At the Spatial Interpolation Comparison exercise 2004 (G. Dubois, 2005) participants were asked to predict gamma dose rates for Germany at 800 validation points using models fitted with 200 training points. One of the data sets (called 'joker' data set) contained 2 training points with very high values. Modeling such variable with conventional geostatistics is a cumbersome, especially within a fully automated geostatistical interpolation framework such the one implemented in the intamap package (Pebesma et al., 2011):

```
library(intamap)
library(gstat)
data(sic2004)
coordinates(sic.val) <- ~x+y
sic.val$value <- sic.val$joker
coordinates(sic.test) <- ~x+y
pred.sic2004 <- interpolate(sic.val, sic.test, maximumTime = 90)
```

```
FALSE R 2018-07-24 10:48:31 interpolating 200 observations, 808 prediction locations
FALSE [1] "estimated time for  copula 71.6110222693811"
FALSE Checking object ... OK
```

where `interpolate` is a fully automated framework for spatial predictions that selects from 5--6 state-of-the-art methods (Pebesma et al., 2011). The resulting error at validation points seems to be relatively high, which is probably due to the choice of transformation and/or variogram model:

```
sd(sic.test$joker-pred.sic2004$predictions$mean)
```

```
## [1] 104.2748
```

We can test predicting those values also using RFsp. First, we need to prepare geographical covariates:

```
bbox=sic.val@bbox
bbox[,"min"]=bbox[,"min"]-4000
bbox[,"max"]=bbox[,"max"]+4000
de2km = plotKML::vect2rast(sic.val, cell.size=2000, bbox=bbox)
de2km$mask = 1
de2km = as(de2km["mask"], "SpatialPixelsDataFrame")
de.dist0 <- GSIF::buffer.dist(sic.val["joker"], de2km, as.factor(1:nrow(sic.val@data)))
```

second, we can fit a RFsp model:

```
ov.de = over(sic.val["joker"], de.dist0)
de.dn0 <- paste(names(de.dist0), collapse="+")
de.fm1 <- as.formula(paste("joker ~ ", de.dn0))
de.rm = do.call(cbind, list(sic.val@data["joker"], ov.de))
m1.gamma <- ranger(de.fm1, de.rm[complete.cases(de.rm),], mtry=1)
m1.gamma
```

```
## Ranger result
##
## Call:
##  ranger(de.fm1, de.rm[complete.cases(de.rm), ], mtry = 1)
##
## Type:                             Regression
## Number of trees:                  500
## Sample size:                      200
## Number of independent variables:  200
## Mtry:                             1
## Target node size:                 5
## Variable importance mode:         none
## OOB prediction error (MSE):       13139.21
## R squared (OOB):                  0.1165779
```

these predictions (when evaluated using the validation points) show better accuracy than obtained using the `interpolate` function:

```
de2km$gamma_rfd1 = predict(m1.gamma, de.dist0@data)$predictions
ov.test <- over(sic.test, de2km["gamma_rfd1"])
sd(sic.test$joker-ov.test$gamma_rfd1, na.rm=TRUE)
```

```
## [1] 66.92076
```

this number matches also the average score generated by multiple groups at the SIC 2004 (G. Dubois, 2005). So in summary, although the OOB prediction error for the model above is still relatively high, RFsp manages to produce more accurate predictions than the `interpolate` function, probably because it does better job in accounting for the local hot-spots. Note also we set `mtry=1` here on purpose low because otherwise importance of the individual 1–2 hotspots would drop significantly.

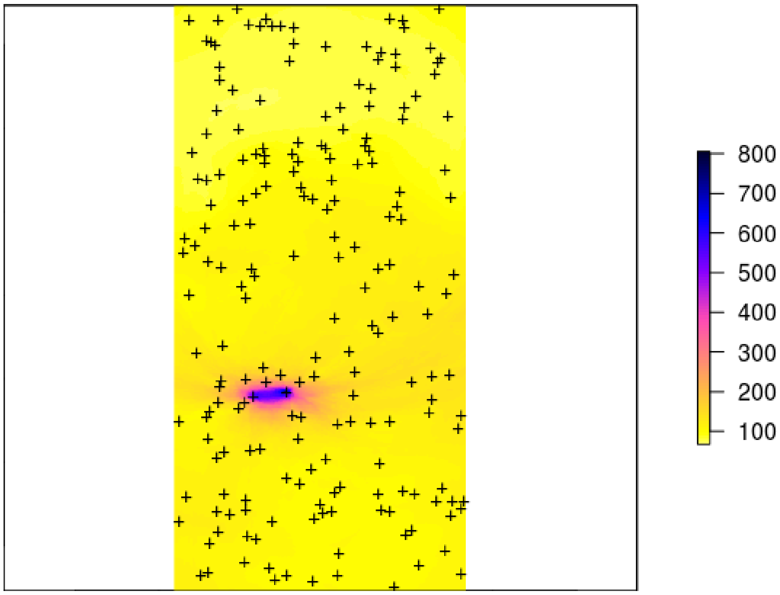

In summary RFsp has a potential to produce maps also for variables with extereme values i.e. very skewed distributions, but this does require that some parameters of RFsp (`mtry`) are carefuly fine-tuned.

Weighted RFsp

In many cases training data sets (points) come with variable measurement errors or have been collected with a sampling bias. Package ranger allows incorporating data quality into the modeling process via the argument `case.weights` — observations with larger weights will be selected with higher probability in the bootstrap, so that the output model will be (correctly) more influenced by observations with higher weights. Consider for example this soil point data set prepared as a combination of (a) the National Cooperative Soil Survey (NCSS) Characterization Database i.e. highly accurate laboratory measurements, and (b) National Soil Information System (NASIS) points i.e. quick observations of soil texture (Ramcharan et al., 2018):

```
carson <- read.csv(file="./RF_vs_kriging/data/NRCS/carson_CLYPPT.csv")
str(carson)
```

```
## 'data.frame':    3418 obs. of  9 variables:
##  $ X.1      : int  1 2 3 4 5 6 8 9 10 11 ...
##  $ SOURCEID : Factor w/ 3230 levels "00CA693X017jbf",..: 1392 1393 3101 3102 3103 3098 3100 893 894 895
...
##  $ pscs     : Factor w/ 25 levels "ASHY","ASHY OVER CLAYEY",..: 19 7 16 16 16 16 16 7 20 20 ...
##  $ X        : num  6372966 6369347 6254691 6254931 6255077 ...
##  $ Y        : num  3737712 3750771 3793830 3793826 3794269 ...
##  $ CLYPPT   : int  20 64 27 27 27 27 27 64 20 20 ...
##  $ CLYPPT.sd: int  8 16 6 6 6 6 6 16 8 8 ...
##  $ SOURCEDB : Factor w/ 2 levels "NASIS","NCSS": 1 1 1 1 1 1 1 1 1 1 ...
##  $ DEPTH    : int  NA NA NA NA NA NA NA NA NA NA ...
```

we focus here on mapping `CLYPPT` i.e. clay content in percent, for which we also would like to use the quality column `CLYPPT.sd` (the standard deviation of the measurement error). The number of NASIS points is of course much higher (ca. 5x) than number of NCSS points, but NCSS points contain about 3x more accurately estimated clay content.

Next we load covariate layers (49), overlay points and grids and prepare a regression matrix:

```
carson$DEPTH.f = ifelse(is.na(carson$DEPTH), 20, carson$DEPTH)
carson1km <- readRDS("./RF_vs_kriging/data/NRCS/carson_covs1km.rds")
coordinates(carson) <- ~X+Y
```

```
proj4string(carson) = carson1km@proj4string
rm.carson <- cbind(as.data.frame(carson), over(carson["CLYPPT"], carson1km))
fm.clay <- as.formula(paste("CLYPPT ~ DEPTH.f + ", paste(names(carson1km), collapse = "+")))
fm.clay
```

```
## CLYPPT ~ DEPTH.f + CRVMRG5_1km + DEMMRG5_1km + DVMMRG5_1km +
##     L01USG5_1km + L02USG5_1km + L03USG5_1km + L04USG5_1km + L05USG5_1km +
##     L06USG5_1km + L07USG5_1km + L08USG5_1km + L09USG5_1km + L10USG5_1km +
##     L11USG5_1km + L13USG5_1km + L14USG5_1km + L15USG5_1km + L16USG5_1km +
##     M01MOD4_1km + M02MOD4_1km + M03MOD4_1km + M04MOD4_1km + M05MOD4_1km +
##     M06MOD4_1km + M07MOD4_1km + M08MOD4_1km + M09MOD4_1km + M10MOD4_1km +
##     M11MOD4_1km + M12MOD4_1km + NEGMRG5_1km + POSMRG5_1km + PRSMRG3_1km +
##     SLPMRG5_1km + T01MOD3_1km + T02MOD3_1km + T03MOD3_1km + T04MOD3_1km +
##     T05MOD3_1km + T06MOD3_1km + T07MOD3_1km + T08MOD3_1km + T09MOD3_1km +
##     T10MOD3_1km + T11MOD3_1km + T12MOD3_1km + TWIMRG5_1km + VBFMRG5_1km +
##     VDPMRG5_1km
```

Note that, because many soil properties are measured at multiple depth, we fit here a 3D spatial prediction model that also takes `DEPTH` into account. This is in fact a rather large data set that we can subset to e.g. 2000 point pairs to speed up computing:

```
rm.carson <- rm.carson[complete.cases(rm.carson[,all.vars(fm.clay)]),]
rm.carson.s <- rm.carson[sample.int(size=2000, nrow(rm.carson)),]
```

Weighted RF model can finally be fitted using:

```
m.clay <- ranger(fm.clay, rm.carson.s, num.trees=150, mtry=25, case.weights=1/(rm.carson.s$CLYPPT.sd^2), qua
m.clay
```

```
## Ranger result
##
## Call:
##  ranger(fm.clay, rm.carson.s, num.trees = 150, mtry = 25, case.weights = 1/(rm.carson.s$CLYPPT.sd^2), quantreg = TRUE)
##
## Type:                             Regression
## Number of trees:                  150
## Sample size:                      2000
## Number of independent variables:  50
## Mtry:                             25
## Target node size:                 5
## Variable importance mode:         none
## OOB prediction error (MSE):       186.608
## R squared (OOB):                  0.2192148
```

in this case we used inverse measurement variance as `case.weights` so that points that were measured in the lab will receive much higher weights. Final output map below shows that, in this specific case, the model without weights seems to predict somewhat higher values, especially in the extrapolation areas. This indicates that using measurement errors in model calibration is important and one should not avoid specifying this in the model, especially if the training data is significantly heterogeneous.

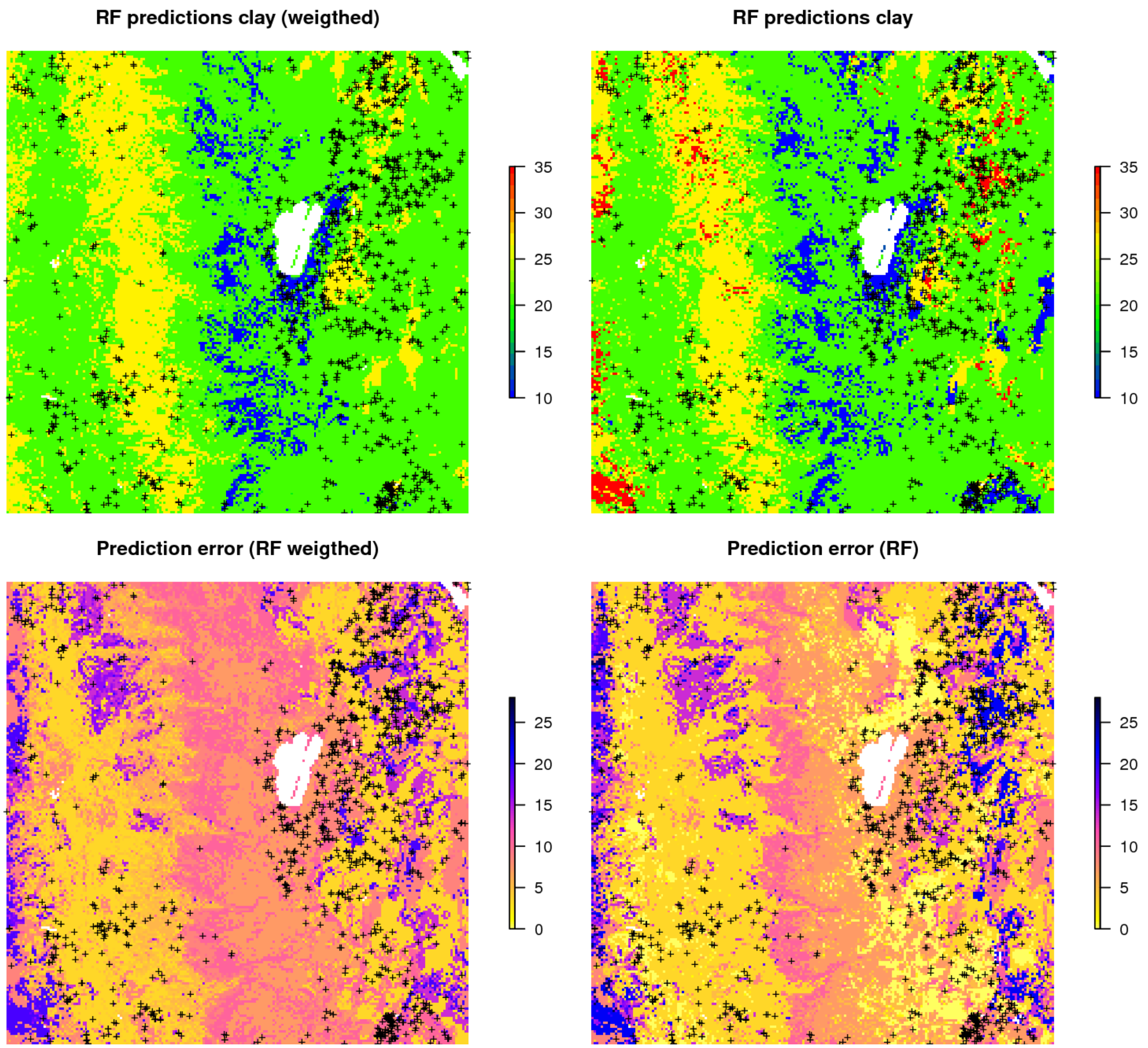

Figure: RF predictions and predictions errors for clay content with and without using measurement errors as weights. Study area around the Lake Tahoe, California USA. Point data sources: National Cooperative Soil Survey (NCSS) Characterization Database and National Soil Information System (NASIS).

Spatial prediction of multivariate problems

Because RF is a decision tree-based method, this opens a possibility to model multiple variables within a single model i.e. by using type of variable as a factor-type covariate. Consider for example the National Geochemical Survey database that contains over 70,000 sampling points spread over the USA (Grossman, Grosz, Schweitzer, & Schruben, 2004). Here we use a subset of this data with 2858 points with measurements of Pb, Cu, K and Mg covering the US states Illinois and Indiana. Some useful covariates to help explain distribution of elements in stream sediments and soils have been previously prepared (Hengl, 2009):

```
geochem = readRDS("./RF_vs_kriging/data/geochem/geochem.rds")
usa5km = readRDS("./RF_vs_kriging/data/geochem/usa5km.rds")
```

```
str(usa5km@data)
```

```
## 'data.frame':    16000 obs. of  6 variables:
##  $ geomap   : Factor w/ 17 levels "6","7","8","13",..: 9 9 9 9 9 9 9 9 9 9 ...
```

```
##  $ globedem : num  266 269 279 269 269 271 284 255 253 285 ...
##  $ dTRI     : num  0.007 0.007 0.008 0.008 0.009 ...
##  $ nlights03: num  6 5 0 5 0 1 5 13 5 5 ...
##  $ dairp    : num  0.035 0.034 0.035 0.036 0.038 ...
##  $ sdroads  : num  0 0 5679 0 0 ...
```

```
## negative values are in fact detection limits:
for(i in c("PB_ICP40","CU_ICP40","K_ICP40","MG_ICP40")) { geochem[,i] = ifelse(geochem[,i] < 0, abs(geochem[
coordinates(geochem) = ~coords.x1 + coords.x2
proj4string(geochem) = "+proj=longlat +ellps=clrk66 +towgs84=-9.0,151.0,185.0,0.0,0.0,0.0,0.0 +no_defs"
geochem$TYPEDESC = as.factor(paste(geochem$TYPEDESC))
summary(geochem$TYPEDESC)
```

```
##         SOIL STRM-SED-DRY STRM-SED-WET      UNKNOWN
##          362          251         2233           12
```

```
geochem = spTransform(geochem, CRS(proj4string(usa5km)))
usa5km.spc = spc(usa5km, ~geomap+globedem+dTRI+nlights03+dairp+sdroads)
```

```
## Converting geomap to indicators...

## Converting covariates to principal components...
```

```
ov.geochem = over(x=geochem, y=usa5km.spc@predicted)
```

we focus on mapping four chemical elements at once:

```
t.vars = c("PB_ICP40","CU_ICP40","K_ICP40","MG_ICP40")
```

in order to model all variables at once, we need to rename them into a single column name that we call `Y` :

```
df.lst = lapply(t.vars, function(i){cbind(geochem@data[,c(i,"TYPEDESC")], ov.geochem)})
names(df.lst) = t.vars
for(i in t.vars){colnames(df.lst[[i]])[1] = "Y"}
for(i in t.vars){df.lst[[i]]$TYPE = i}
```

All variables are now bind into a single column:

```
rm.geochem = do.call(rbind, df.lst)
type.mat = data.frame(model.matrix(~TYPE-1, rm.geochem))
typed.mat = data.frame(model.matrix(~TYPEDESC-1, rm.geochem))
```

The final regression matrix is thus:

```
rm.geochem.e = do.call(cbind, list(rm.geochem[,c("Y",paste0("PC",1:21))], type.mat, typed.mat))
```

so that we can fit a single model for four chemical elements:

```
fm.g = as.formula(paste0("Y ~ ", paste0(names(rm.geochem.e)[-1], collapse = "+")))
fm.g
```

```
## Y ~ PC1 + PC2 + PC3 + PC4 + PC5 + PC6 + PC7 + PC8 + PC9 + PC10 +
##     PC11 + PC12 + PC13 + PC14 + PC15 + PC16 + PC17 + PC18 + PC19 +
##     PC20 + PC21 + TYPECU_ICP40 + TYPEK_ICP40 + TYPEMG_ICP40 +
##     TYPEPB_ICP40 + TYPEDESCSOIL + TYPEDESCSTRM.SED.DRY + TYPEDESCSTRM.SED.WET +
##     TYPEDESCUNKNOWN
```

```
m1.geochem <- ranger::ranger(fm.g, rm.geochem.e[complete.cases(rm.geochem.e),], importance = "impurity", see
m1.geochem
```

```
## Ranger result
##
## Call:
##  ranger::ranger(fm.g, rm.geochem.e[complete.cases(rm.geochem.e),      ], importance = "impurity", seed
= 1)
##
## Type:                             Regression
## Number of trees:                  500
## Sample size:                      11148
## Number of independent variables:  29
## Mtry:                             5
## Target node size:                 5
## Variable importance mode:         impurity
## OOB prediction error (MSE):       1446.878
## R squared (OOB):                  0.4041142
```

where `TYPECU_ICP40` is the indicator variable specifying which values `Y` refer to `CU_ICP40` i.e. copper contration. This single multivariate model can now be used to predict any of the four chemicals in combination with any `TYPEDESC` .

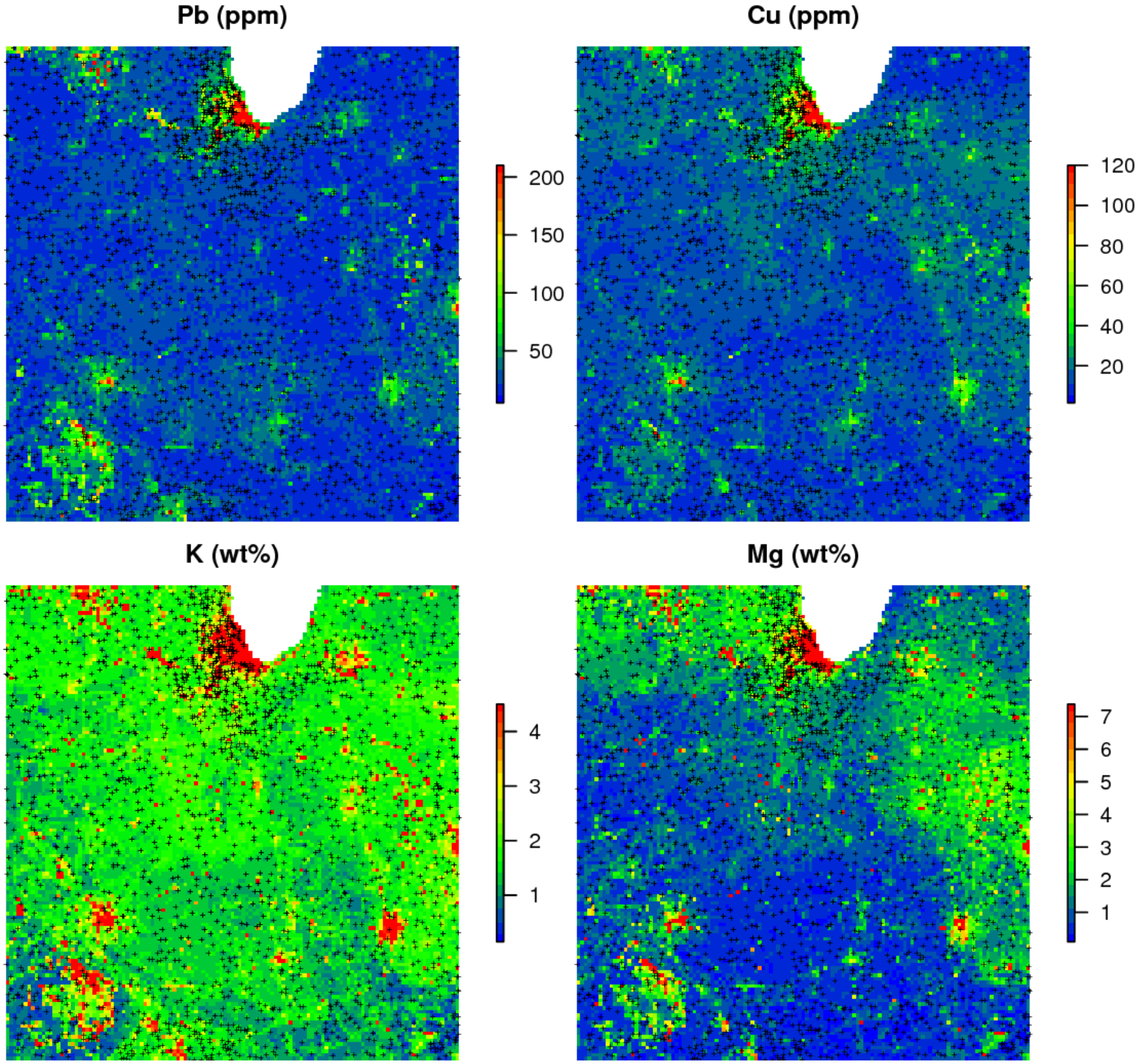

Figure: Predictions produced for four chemical elements (wet stream sediments) from the National Geochemical Survey using a single multivariate RF model. Area covers the US States Illinois and Indiana. Spatial resolution of predictions is 5 km.

A disadvantage of running multivariate models is that the data size increases rapidly as the number of target variables increases and hence also the computing intensity increases exponentially. For a comparison, the National Geochemical Survey comprises hundreds of chemical elements hence the total size of training points could easily exceed several millions. In addition, further model diagnostics such as variable importance and similar becomes difficult as all variables are included in a single model — indicates overall R-square of 0.40, but not all chemical elements can be mapped with the same accuracy. Note also that in this case study we do not derive any buffer distances (also because the sampling points are well distributed and this data set is rich with covariates) as this would blow up computing load exponentially.

Prediction of spatio-temporal variable

In the last example we look at extending 2D regression based on RFsp to a spatiotemporal data i.e. to a 2D+T case. For this we use a time-series of daily precipitation measurements obtained from https://www.ncdc.noaa.gov for the period 2014–2017 for the area around Boulder Colorado:

```
co_prec = readRDS("./RF_vs_kriging/data/st_prec/boulder_prcp.rds")
str(co_prec)
```

```
## 'data.frame':    176467 obs. of  16 variables:
##  $ STATION  : Factor w/ 239 levels "US1COBO0004",..: 64 64 64 64 64 64 64 64 64 64 ...
##  $ NAME     : Factor w/ 233 levels "ALLENS PARK 1.5 ESE, CO US",..: 96 96 96 96 96 96 96 96 96 96 ...
##  $ LATITUDE : num  40.1 40.1 40.1 40.1 40.1 ...
##  $ LONGITUDE: num  -105 -105 -105 -105 -105 ...
##  $ ELEVATION: num  1567 1567 1567 1567 1567 ...
##  $ DATE     : Factor w/ 1462 levels "2014-11-01","2014-11-02",..: 7 13 21 35 46 67 68 69 70 75 ...
##  $ PRCP     : num  0 0.16 0 0 0 0.01 0.02 0.02 0.02 0.01 ...
##  $ SNOW     : num  0 NA 0 0 0 NA NA NA NA 0 ...
##  $ SNWD     : int  NA NA NA NA NA NA NA NA NA NA ...
##  $ TAVG     : int  NA NA NA NA NA NA NA NA NA NA ...
##  $ WT01     : int  NA NA NA NA NA NA NA NA NA NA ...
##  $ WT03     : int  NA NA NA NA NA NA NA NA NA NA ...
##  $ WT04     : int  NA NA NA NA NA NA NA NA NA NA ...
##  $ WT05     : int  NA NA NA NA NA NA NA NA NA NA ...
##  $ WT06     : int  NA NA NA NA NA NA NA NA NA NA ...
##  $ WT11     : int  NA NA NA NA NA NA NA NA NA NA ...
```

this is a relatively large data set with almost 200,000 observations (such spatiotemporal data sets are typically large). Next we derive cumulative day (`cdate`) and day of the year (`doy`) which we will also use further as covariates to explain daily precipitation:

```
co_prec$cdate = floor(unclass(as.POSIXct(as.POSIXct(paste(co_prec$DATE), format="%Y-%m-%d")))/86400)
co_prec$doy = as.integer(strftime(as.POSIXct(paste(co_prec$DATE), format="%Y-%m-%d"), format = "%j"))
```

We also extract fixed spatial locations of stations and overaly with covariates such as elevation (`elev_1km`) and long-term precipitation maps (`PRISM_prec`):

```
co_locs.sp = co_prec[!duplicated(co_prec$STATION),c("STATION","LATITUDE","LONGITUDE")]
coordinates(co_locs.sp) = ~ LONGITUDE + LATITUDE
proj4string(co_locs.sp) = CRS("+proj=longlat +datum=WGS84")
co_grids = readRDS("./RF_vs_kriging/data/st_prec/boulder_grids.rds")
co_grids = as(co_grids, "SpatialPixelsDataFrame")
co_locs.sp = spTransform(co_locs.sp, co_grids@proj4string)
sel.co <- over(co_locs.sp, co_grids[1])
co_locs.sp <- co_locs.sp[!is.na(sel.co$elev_1km),]
```

and derive buffer distances for fixed stations:

```
grid.distP <- GSIF::buffer.dist(co_locs.sp["STATION"], co_grids[1], as.factor(1:nrow(co_locs.sp)))
dnP <- paste(names(grid.distP), collapse="+")
```

so that we can define a *hybrid* space-time model that can be used to predict daily rainfall as a function of time, covariates and buffer distances (i.e. geographical space):

```
fmP <- as.formula(paste("PRCP ~ cdate + doy + elev_1km + PRISM_prec +", dnP))
fmP
```

```
## PRCP ~ cdate + doy + elev_1km + PRISM_prec + layer.1 + layer.2 +
##     layer.3 + layer.4 + layer.5 + layer.6 + layer.7 + layer.8 +
##     layer.9 + layer.10 + layer.11 + layer.12 + layer.13 + layer.14 +
##     layer.15 + layer.16 + layer.17 + layer.18 + layer.19 + layer.20 +
##     layer.21 + layer.22 + layer.23 + layer.24 + layer.25 + layer.26 +
##     layer.27 + layer.28 + layer.29 + layer.30 + layer.31 + layer.32 +
##     layer.33 + layer.34 + layer.35 + layer.36 + layer.37 + layer.38 +
##     layer.39 + layer.40 + layer.41 + layer.42 + layer.43 + layer.44 +
##     layer.45 + layer.46 + layer.47 + layer.48 + layer.49 + layer.50 +
##     layer.51 + layer.52 + layer.53 + layer.54 + layer.55 + layer.56 +
##     layer.57 + layer.58 + layer.59 + layer.60 + layer.61 + layer.62 +
##     layer.63 + layer.64 + layer.65 + layer.66 + layer.67 + layer.68 +
##     layer.69 + layer.70 + layer.71 + layer.72 + layer.73 + layer.74 +
```

```
##     layer.75 + layer.76 + layer.77 + layer.78 + layer.79 + layer.80 +
##     layer.81 + layer.82 + layer.83 + layer.84 + layer.85 + layer.86 +
##     layer.87 + layer.88 + layer.89 + layer.90 + layer.91 + layer.92 +
##     layer.93 + layer.94 + layer.95 + layer.96 + layer.97 + layer.98 +
##     layer.99 + layer.100 + layer.101 + layer.102 + layer.103 +
##     layer.104 + layer.105 + layer.106 + layer.107 + layer.108 +
##     layer.109 + layer.110 + layer.111 + layer.112 + layer.113 +
##     layer.114 + layer.115 + layer.116 + layer.117 + layer.118 +
##     layer.119 + layer.120 + layer.121 + layer.122 + layer.123 +
##     layer.124 + layer.125 + layer.126 + layer.127 + layer.128 +
##     layer.129 + layer.130 + layer.131 + layer.132 + layer.133 +
##     layer.134 + layer.135 + layer.136 + layer.137 + layer.138 +
##     layer.139 + layer.140 + layer.141 + layer.142 + layer.143 +
##     layer.144 + layer.145 + layer.146 + layer.147 + layer.148 +
##     layer.149 + layer.150 + layer.151 + layer.152 + layer.153 +
##     layer.154 + layer.155 + layer.156 + layer.157 + layer.158 +
##     layer.159 + layer.160 + layer.161 + layer.162 + layer.163 +
##     layer.164 + layer.165 + layer.166 + layer.167 + layer.168 +
##     layer.169 + layer.170 + layer.171 + layer.172 + layer.173 +
##     layer.174 + layer.175 + layer.176 + layer.177 + layer.178 +
##     layer.179 + layer.180 + layer.181 + layer.182 + layer.183 +
##     layer.184 + layer.185 + layer.186 + layer.187 + layer.188 +
##     layer.189 + layer.190 + layer.191 + layer.192 + layer.193 +
##     layer.194 + layer.195 + layer.196 + layer.197 + layer.198 +
##     layer.199 + layer.200 + layer.201 + layer.202 + layer.203 +
##     layer.204 + layer.205 + layer.206 + layer.207 + layer.208 +
##     layer.209 + layer.210 + layer.211 + layer.212 + layer.213 +
##     layer.214 + layer.215 + layer.216 + layer.217 + layer.218 +
##     layer.219 + layer.220 + layer.221 + layer.222 + layer.223 +
##     layer.224 + layer.225
```

To produce the space-time regression matrix we bind together buffer distances, values of covariates and time-variables:

```
ov.prec <- do.call(cbind, list(co_locs.sp@data, over(co_locs.sp, grid.distP), over(co_locs.sp, co_grids[c("e
rm.prec <- plyr::join(co_prec, ov.prec)
```

```
## Joining by: STATION
```

```
rm.prec <- rm.prec[complete.cases(rm.prec[,c("PRCP","elev_1km","cdate")]),]
```

Further fitting of RFsp for this spacetime data follows the same framework as used in all other examples previously. Based on this model, we can generate predictions through the space-time domain of interest, which typically results in producing time-series of raster maps as indicated below.

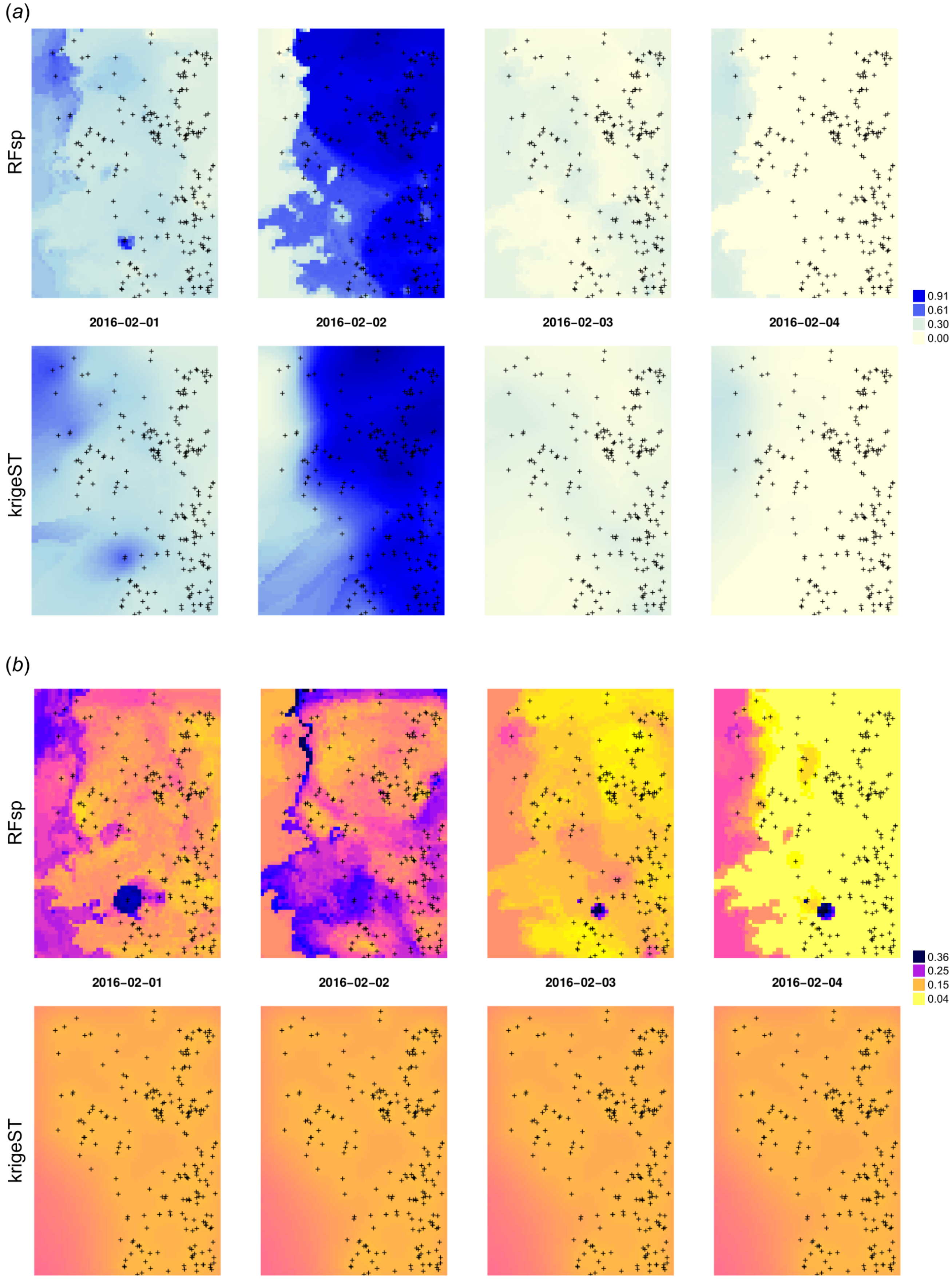

Figure: Spatiotemporal observations (points) and predictions of daily rainfall in mm for four day in February using the RFsp and krigeST methods: (a) predictions, (b) standard deviation of prediction errors estimated using the ranger package.

Note from the maps above that some hot spots in the prediction error maps from previous days might propagate to other days, which indicates spatiotemporal connection between values. This indicates spatiotemporal connection between values in the output predictions.

One disadvantage of fitting spatiotemporal models using station data is that the actual accuracy of this models need to be assessed using leave-locations-out cross-validation (Meyer, Reudenbach, Hengl, Katurji, & Nauss, 2018), otherwise ranger might give an overoptimistic estimate of the actual accuracy. This happens because RF learns also from *"location"* so that the realistic estimate of accuracy can often be significantly lower if this issue is ignored.

In summary, Random Forest seems to be suitable for generating spatial and spatiotemporal predictions once the geographical distances are added to the model (see also (Behrens et al., n.d.)). Computing time, however, can be a cumbersome and working with data sets with >>1000 point locations (hence >>1000 buffer distance maps) is problably not yet recommended. Also cross-validation of accuracy of predictions produced using RFsp needs to be implemented using leave-location-out CV to account for spatial autocorrelation in data. The key to the success of the RFsp framework might be the training data quality — especially quality of spatial sampling (to minimize extrapolation problems and any type of bias in data), and quality of model validation (to ensure that accuracy is not effected by overfitting). For all other details please refer to our paper.

References

Behrens, T., Schmidt, K., Viscarra Rossel, R. A., Gries, P., Scholten, T., & MacMillan, R. A. (n.d.). Spatial modelling with euclidean distance fields and machine learning. *European Journal of Soil Science*, *in press*. doi:10.1111/ejss.12687

Brown, P. E. (2015). Model-based geostatistics the easy way. *Journal of Statistical Software*, *63*(12). Retrieved from http://www.jstatsoft.org/v63/i12

Diggle, P. J., & Ribeiro Jr, P. J. (2007). *Model-based Geostatistics* (p. 288). Springer.

Dubois, G. (Ed.). (2005). *Automatic mapping algorithms for routine and emergency monitoring data* (p. 150). Luxembourg: Office for Official Publications of the European Communities.

Dubois, G., Malczewski, J., & De Cort, M. (2003). *Mapping radioactivity in the environment: Spatial interpolation comparison 97*. Office for Official Publications of the European Communities.

Grossman, J. N., Grosz, A. E., Schweitzer, P. N., & Schruben, P. G. (2004). *The National Geochemical Survey-database and documentation*. USGS Eastern Mineral; Environmental Resources Science Center.

Hengl, T. (2009). *A practical guide to geostatistical mapping*. Amsterdam, the Netherlands: Lulu.

Hijmans, R. J., & Etten, J. van. (2017). *Raster: Geographic data analysis and modeling*. Retrieved from https://cran.r-project.org/package=raster

Meinshausen, N. (2006). Quantile regression forests. *Journal of Machine Learning Research*, *7*, 983–999.

Meyer, H., Reudenbach, C., Hengl, T., Katurji, M., & Nauss, T. (2018). Improving performance of spatio-temporal machine learning models using forward feature selection and target-oriented validation. *Environmental Modelling & Software*, *101*, 1–9. doi:10.1016/j.envsoft.2017.12.001

Pebesma, E., Cornford, D., Dubois, G., Heuvelink, G. B., Hristopulos, D., Pilz, J., … Skøien, J. O. (2011). INTAMAP: The design and implementation of an interoperable automated interpolation web service. *Computers & Geosciences*, *37*(3), 343–352.

Pekel, J.-F., Cottam, A., Gorelick, N., & Belward, A. S. (2016). High-resolution mapping of global surface water and its long-term changes. *Nature*, *504*, 418–422.

Ramcharan, A., Hengl, T., Nauman, T., Brungard, C., Waltman, S., Wills, S., & Thompson, J. (2018). Soil property and class maps of the conterminous us at 100 meter spatial resolution based on a compilation of national soil point observations and machine learning. *Soil Science Society of America Journal*, *82*, 186–201. doi:10.2136/sssaj2017.04.0122

Wager, S., Hastie, T., & Efron, B. (2014). Confidence intervals for random forests: The jackknife and the infinitesimal jackknife. *Journal of Machine Learning Research*, *15*(1), 1625–1651.

Wright, M. N., & Ziegler, A. (2017). ranger: A Fast Implementation of Random Forests for High Dimensional Data in C++ and R. *Journal of Statistical Software*, *77*(1), 1–17.
